# Comparison of post-acute sequelae following hospitalization for COVID-19 and influenza

**DOI:** 10.1186/s12916-023-03200-2

**Published:** 2023-12-05

**Authors:** Ting-Hui Liu, Po-Yu Huang, Jheng-Yan Wu, Min-Hsiang Chuang, Wan-Hsuan Hsu, Ya-Wen Tsai, Chih-Cheng Lai

**Affiliations:** 1 Department of Psychiatry, Chi Mei Medical Center, Tainan, Taiwan; 2 Department of Internal Medicine, Chi Mei Medical Center, Tainan, Taiwan; 3 Department of Nutrition, Chi Mei Medical Center, Tainan, Taiwan; 4 Center for Integrative Medicine, Chi Mei Medical Center, Tainan, Taiwan; 5 Division of Hospital Medicine, Department of Internal Medicine, Chi Mei Medical Center, No 901, Chunghwa Road, Yongkang District, Tainan City 710, Taiwan; 6 School of Medicine, College of Medicine, National Sun Yat-Sen University, Kaohsiung, Taiwan

**Keywords:** COVID-19, Influenza, Post-COVID condition, SARS-CoV-2

## Abstract

**Background:**

Few studies have directly compared the risk and magnitude of post-acute sequelae following COVID-19 and influenza, and most of these studies were conducted before emergence of the Omicron. This study investigated the prevalence of post-COVID conditions and the long-term risk of emergency department (ED) visits, hospitalizations, and deaths in patients with COVID-19 and compared their risk with that of patients with influenza.

**Methods:**

A retrospective study based on the TriNetX databases, a global health research network. We identified patients with COVID-19 and influenza who required hospitalization between January 1, 2022, and January 1, 2023. We compared the risk of developing any post-COVID conditions between the two groups and also analyzed each post-COVID-19 condition and all-cause ED visits, hospitalizations, and deaths in both populations during the follow-up 90–180 days.

**Results:**

Before matching, 7,187 patients with COVID-19 were older (63.9 ± 16.7 vs. 55.4 ± 21.2) and were predominantly male (54.0% vs. 45.4%), and overweight/obese (16.1% vs. 11.2%) than 11,266 individuals with influenza. After propensity score matching, 6,614 patients were identified in each group, resulting in well-balanced baseline characteristics. During follow-up, the COVID-19 group had a higher incidence of any post-COVID-19 condition when compared with the influenza group (17.9% vs. 13.0%), with a hazard ratio (HR) of 1.398 (95% CI, 1.251–1.562). Compared to the influenza group, the COVID-19 group had a significantly higher incidence of abnormal breathing (HR, 1.506; 95% CI, 1.246–1.822), abdominal symptoms (HR, 1.313; HR, 1.034–1.664), fatigue (HR, 1.486; 95% CI, 1.158–1.907), and cognitive symptoms (HR, 1.815; 95% CI, 1.235–2.668). Moreover, the COVID-19 group had a significantly higher risk of the composite outcomes during all-cause ED visits, hospitalizations, and deaths when compared with the influenza group (27.5% vs. 21.7; HR, 1.303; 95% CI, 1.194–1.422).

**Conclusions:**

This study indicates that hospitalized COVID-19 patients are at a higher risk of long-term complications when compared with influenza survivors.

**Supplementary Information:**

The online version contains supplementary material available at 10.1186/s12916-023-03200-2.

## Background

The COVID-19 pandemic has brought unprecedented challenges to global health systems, economies, and societies since its first outbreak at the end of 2019 [1, 2]. Over the past few years, the world has experienced the devastating impact of SARS-CoV-2, which includes more than 767 million COVID-19 cases and 6,947,192 deaths as of June 28, 2023 [3]. Fortunately, the rapid development of vaccines against SARS-CoV-2 has provided individuals with enhanced protection against severe illness and hospitalization [4–7]. Additionally, the availability of antiviral treatments has further enhanced patient care and management [8]. Antiviral drugs, such as nirmatrelvir plus ritonavir, molnupiravir, and remdesivir, have shown promising results in preventing the progression of non-hospitalized patients with COVID-19 [8–12]. Remdesivir has also been shown to reduce the recovery duration of hospitalized patients with severe COVID-19 [13]. The combined efforts in vaccine development, availability of antiviral treatments, and public health measures have been crucial in controlling the spread of the virus, protecting vulnerable populations, and improving the overall outcomes of patients affected by COVID-19. Therefore, the World Health Organization declared that we should cautiously return to a pre-pandemic state [14].

However, it is crucial to recognize that the aftermath of COVID-19 may extend beyond the acute phase, giving rise to a condition commonly referred to as "long COVID" or "post-COVID conditions" [15, 16]. Post-COVID conditions encompass a range of persistent symptoms and complications that individuals experience after recovering from the acute phase of COVID-19. These symptoms can persist for weeks or even months, significantly affecting the quality of life and overall well-being of the affected individuals [17]. Common manifestations of post-COVID conditions include fatigue, dyspnea, cognitive impairment, cardiac abnormalities, and psychological distress [18]. As the number of individuals surviving COVID-19 hospitalization continues to rise, understanding the long-term consequences of the disease becomes crucial for providing appropriate care and support to affected individuals.

Although the impact of post-COVID conditions has garnered significant attention, it is essential to contextualize this condition by comparing SARS-CoV-2 with other respiratory infections. Influenza is a respiratory virus that poses a threat to public health. However, few studies have directly compared the risk and magnitude of post-acute sequelae following COVID-19 and influenza, and most of these studies were conducted before the emergence of the Omicron [19–23]. Therefore, this study was conducted to compare the risk of post-acute sequelae within 90 to 180 days following hospitalization for COVID-19 and influenza, and to provide updated information for further evidence-based decision-making and public health strategies.

## Methods

### Data source

The present study used the TriNetX COVID-19 Network, an international collaboration of health research platforms that compiles de-identified patient data from electronic health records (EHRs). These records encompass a wide variety of patient information, including demographic details, medical diagnoses, procedures, medication records, laboratory results, genomic data, and types of healthcare organization visits. Over 120 healthcare organizations (HCOs) worldwide, predominantly academic health centers, have contributed data from their main hospitals, affiliated institutions, and outpatient clinics to TriNetX. For this specific analysis, we utilized the COVID-19 network, encompassing data from more than 114 million patients from 87 HCOs. TriNetX offers integrated tools for patient-level data analysis and delivers aggregated results to the researchers. Detailed information on the database can be accessed online [24]. Written informed consent was not required because TriNetX contains anonymized data. The Institutional Review Board of the Chi Mei Medical Center approved the study protocol (no. 11202–002).

### Patient selection

In the patient selection process, the TriNetX database was used, which contains 86 HCOs as of July 4, 2023. The initial patient pool consisted of individuals who had visited these HCOs at least twice between March 1, 2020, and January 1, 2023. Patients who tested positive for SARS-CoV-2 or were diagnosed with COVID-19 between January 1, 2022, and January 1, 2023, and those who were prescribed antiviral agents and were initially hospitalized were identified from this pool. The prescribed antiviral medications included molnupiravir, remdesivir, and nirmatrelvir plus ritonavir. The selection process was identical for all patients diagnosed with influenza within the same timeframe. The analysis was restricted to patients aged ≥ 18.

Subsequently, several exclusion criteria were used. For the COVID-19 group, patients who were also diagnosed with influenza and those with long-term COVID-related symptoms one year before the index date were excluded. Similarly, for the influenza group, patients who tested positive for SARS-CoV-2 or were diagnosed with COVID-19 and those with long-term COVID-related symptoms one year before the index date were excluded.

Finally, patient selection involved propensity score matching on a 1:1 basis for age at index, race, sex, adverse socioeconomic determinants of health, and comorbid medical conditions. This resulted in two comparable groups for this study: a COVID-19 and an influenza group (Tables S1 and S2).

### Covariates

We considered 47 variables to adjust for imbalances in baseline characteristics between the COVID-19 and influenza groups. The list included both confirmed and suspected risk factors for COVID-19 and more severe cases of the illness, such as demographics (eg, age, sex, and ethnicity), adverse socioeconomic determinants of health (including "problems related to education and literacy," "problems related to employment and unemployment," and "problems related to housing and economic circumstances," as defined by ICD-10), and comorbidities (such as obesity, hyperlipidemia, hypertension, diabetes mellitus, chronic kidney disease, asthma, chronic lower respiratory diseases, ischemic heart disease, neoplasm, chronic liver diseases, stroke, dementia, rheumatoid arthritis, lupus, psoriasis, human immunodeficiency virus infection, mood disorders, and psychotic disorders).

### Outcome measurement

The primary outcome of this study was a composite outcome consisting of 12 clinical features of post-COVID conditions, observed 90–180 days after the index event. These features include chest/throat pain, abnormal breathing, abdominal symptoms, fatigue/malaise, anxiety/depression, pain, headache, cognitive dysfunction, myalgia, loss of taste or smell, sleep disturbances, coughing, and palpitations [25–27].

We investigated the secondary outcomes of all-cause hospitalization, all-cause emergency department (ED) visits, and deaths during the follow-up period. Table S3 provides additional details regarding the diagnostic, visiting, and procedural codes used to define these outcomes [21, 28, 29].

### Statistical analysis

We used the built-in propensity score-matching (PSM) function of the TriNetX platform to ensure a 1:1 match between the participants in the COVID-19 and influenza groups. This was achieved using a nearest-neighbor greedy matching algorithm with a caliper width of 0.1 pooled standard deviation. Standard differences were then computed to assess the balance between groups, with differences in absolute values < 0.1, indicating a good match between groups [30].

Subsequently, we performed Kaplan–Meier analysis, followed by log-rank tests and calculation of hazard ratio (HR) with 95% confidence intervals (CI) to compare the two groups. Statistical significance was set at p < 0.05. The HR was used to describe the relative risk of post-COVID conditions based on a comparison of time-to-event rates calculated using a proportional hazard model, which is a built-in function in TriNetX.

For subgroup analysis, we compared the primary and secondary outcomes between the two groups, stratified by age (18–64 and ≥ 65 years), sex, vaccine status (unvaccinated, 1 or 2 doses of vaccine, boosted), and race (Caucasian and non-Caucasian). The vaccine type used was Pfizer with CPT code 91,307 (0051A, 0052A, 0071A, 0072A), Janssen 91,303 (0031A), Novavax (0041A, 0042A), and Moderna 91,301 (0011A, 0012A, 0013A, 0111A).

## Results

### Baseline characteristics

**Fig. 1:**
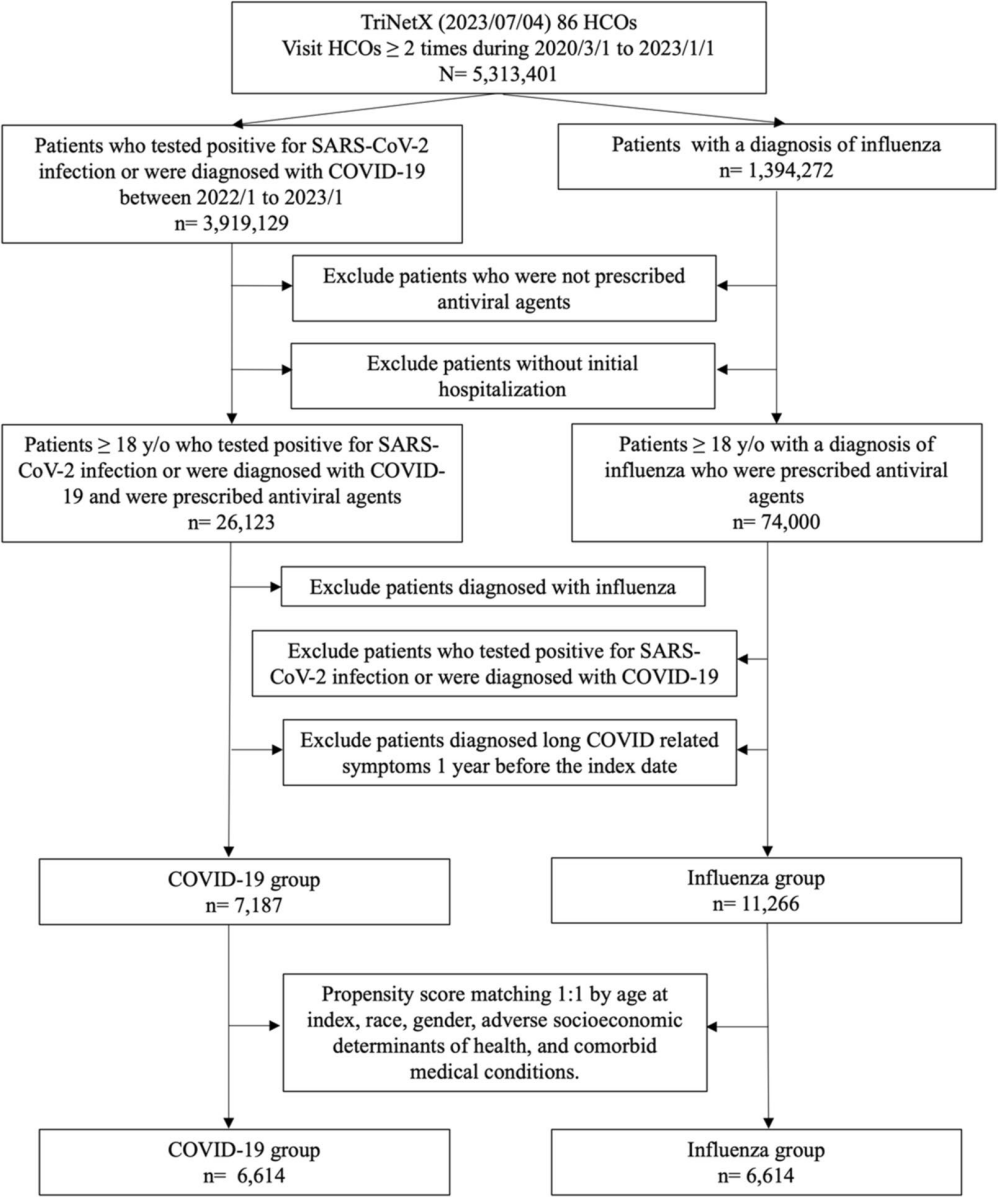
Flowchart of patient selection and construction of the cohort

After excluding ineligible participants, the final cohort comprised 7,187 individuals hospitalized for COVID-19 and 11,266 individuals who survived hospitalization for influenza, serving as the comparator cohorts (Fig. 1). Baseline characteristics differed significantly between the COVID-19 and influenza groups. Patients with COVID-19 were, on average, older compared to those with influenza (63.9 ± 16.7 vs. 55.4 ± 21.2). Furthermore, there were variations in the distribution of sex and race between the two groups (Table 1). The study group also exhibited a higher prevalence of being overweight and obese than the control group (16.1% vs. 11.2%). Conversely, the prevalence of asthma was higher in the influenza group than that in the COVID-19 group (11.0% vs. 5.8%). However, after propensity score matching, 6,614 patients were identified in each group, resulting in well-balanced baseline characteristics. No significant differences were observed between the COVID-19 and influenza groups (Table 1).

**Table 1:** Comparison of characteristics of COVID-19 group and Influenza group before and after matching

	Before matching			After matching		
	COVID-19 group (*n* = 7,187)	Influenza group (*n* = 11,266)	Std diff	COVID-19 group (*n* = 6,614)	Influenza group (*n* = 6,614)	Std diff
**Age at Index** ±	63.9 ± 16.7	55.4 ± 21.2	0.445	62.7 ± 16.7	63.1 ± 17.9	0.019
**Sex**						
Male	3878 (53.96)	5110 (45.36)	0.173	3468 (52.43)	3433 (51.91)	0.011
Female	3308 (46.03)	6155 (54.63)	0.173	3145 (47.55)	3181 (48.1)	0.011
**Race**						
White	4654 (64.76)	6387 (56.69)	0.166	4204 (63.56)	4226 (63.9)	0.007
Asian	194 (2.7)	257 (2.28)	0.027	172 (2.6)	159 (2.4)	0.013
Black or African American	1041 (14.48)	1813 (16.09)	0.045	975 (14.74)	989 (14.95)	0.006
Unknown Race	1204 (16.75)	2718 (24.13)	0.184	1186 (17.93)	1164 (17.6)	0.009
**Socioeconomic determinants of health**						
Problems related to housing and economic circumstances	73 (1.02)	77 (0.68)	0.036	62 (0.94)	55 (0.83)	0.011
Problems related to employment and unemployment	18 (0.25)	21 (0.19)	0.014	16 (0.24)	15 (0.23)	0.003
**Comorbidities**						
Hypertensive diseases	2504 (34.84)	3581 (31.79)	0.065	2322 (35.11)	2330 (35.23)	0.003
Hyperlipidemia	1734 (24.13)	2435 (21.61)	0.060	1602 (24.22)	1589 (24.03)	0.005
Type 2 diabetes mellitus	1359 (18.91)	1762 (15.64)	0.087	1238 (18.72)	1205 (18.22)	0.013
Overweight and obesity	1156 (16.09)	1265 (11.23)	0.142	993 (15.01)	962 (14.55)	0.013
Neoplasms	917 (12.76)	1617 (14.35)	0.047	873 (13.2)	888 (13.43)	0.007
Cerebral infarction	178 (2.48)	215 (1.91)	0.039	151 (2.28)	149 (2.25)	0.002
Ischemic heart diseases	915 (12.73)	1169 (10.38)	0.074	840 (12.7)	840 (12.7)	< 0.001
Substance use disorder	801 (11.15)	1522 (13.51)	0.072	765 (11.57)	774 (11.7)	0.004
Nicotine dependence	626 (8.71)	1186 (10.53)	0.062	598 (9.04)	609 (9.21)	0.006
Mood disorders	610 (8.49)	1116 (9.91)	0.049	570 (8.62)	568 (8.59)	0.001
Chronic kidney disease	770 (10.71)	1005 (8.92)	0.060	706 (10.67)	667 (10.09)	0.019
Chronic lower respiratory diseases						
Asthma	420 (5.84)	1240 (11.01)	0.187	413 (6.24)	405 (6.12)	0.005
Bronchitis	80 (1.11)	240 (2.13)	0.081	77 (1.16)	119 (1.8)	0.053
Emphysema	193 (2.69)	304 (2.7)	0.001	185 (2.8)	184 (2.78)	0.001
Chronic obstructive pulmonary disease	734 (10.21)	1204 (10.69)	0.015	703 (10.63)	703 (10.63)	< 0.001
Chronic liver diseases						
Alcoholic liver disease	34 (0.47)	56 (0.5)	0.003	33 (0.5)	30 (0.45)	0.007
Fibrosis and cirrhosis of liver	80 (1.11)	135 (1.2)	0.008	75 (1.13)	76 (1.15)	0.001
Fatty liver	167 (2.32)	177 (1.57)	0.054	147 (2.22)	137 (2.07)	0.010
Immune disorders						
Sarcoidosis	10 (0.14)	32 (0.28)	0.032	10 (0.15)	10 (0.15)	< 0.001
Human immunodeficiency virus disease	76 (1.06)	120 (1.07)	0.001	69 (1.04)	68 (1.03)	0.001
Immunodeficiency with predominantly antibody defects	18 (0.25)	45 (0.4)	0.026	18 (0.27)	18 (0.27)	< 0.001
Rheumatoid arthritis	88 (1.22)	177 (1.57)	0.030	87 (1.32)	79 (1.19)	0.011
Psoriasis	52 (0.72)	102 (0.91)	0.020	46 (0.7)	53 (0.8)	0.012
Systemic lupus erythematosus	25 (0.35)	72 (0.64)	0.042	25 (0.38)	26 (0.39)	0.002

### Primary outcomes

**Fig. 2:**
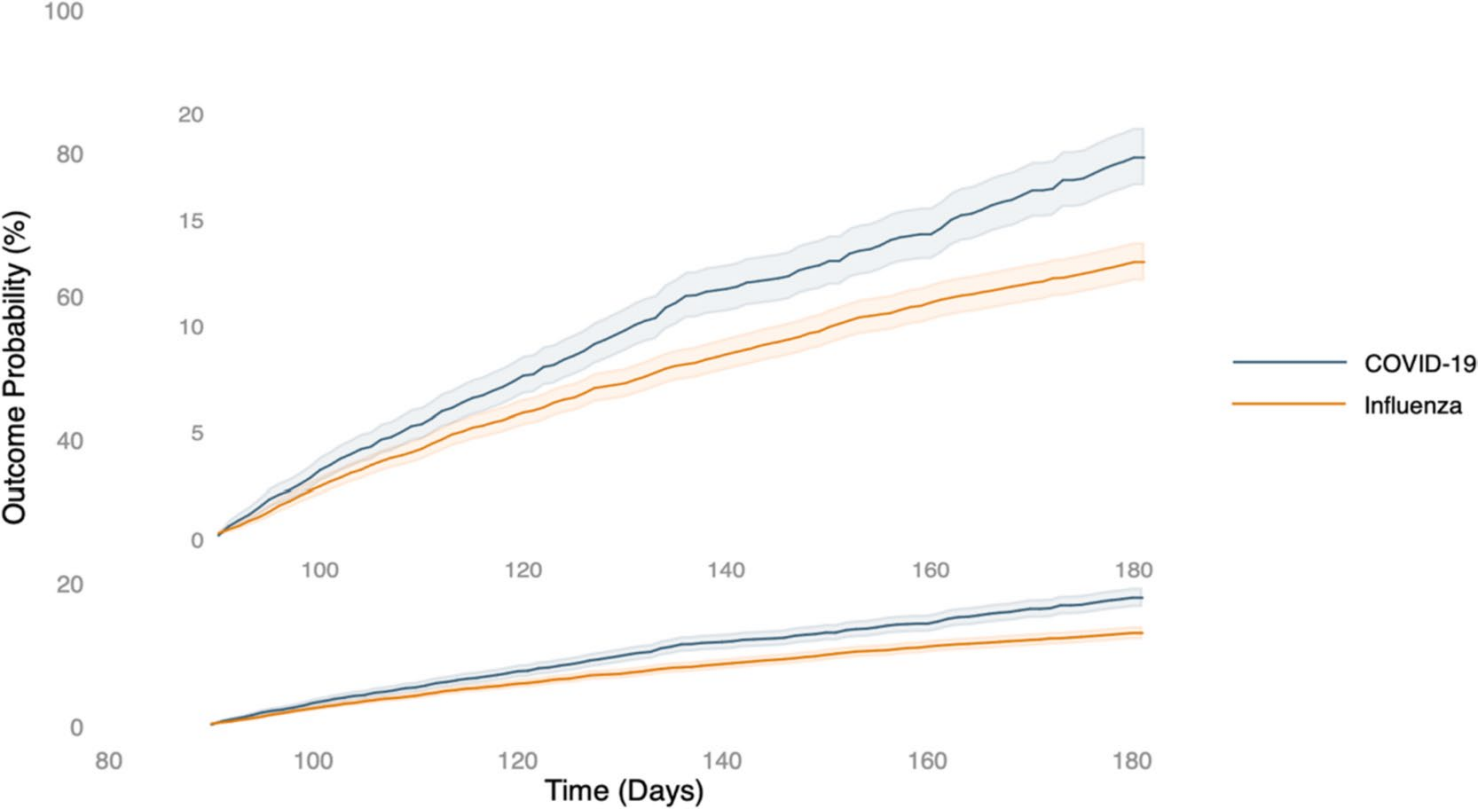
The probability of the primary outcome: a composite of any post-COVID conditions. This figure incorporates two Kaplan–Meier curves with different scales. The lower curve displays the range from 0 to 100%, while the upper curve provides a magnified view from 0 to 20% for more detailed observation of variations. The blue curve represents COVID-19, and the orange curve indicates Influenza

**Fig. 3:**
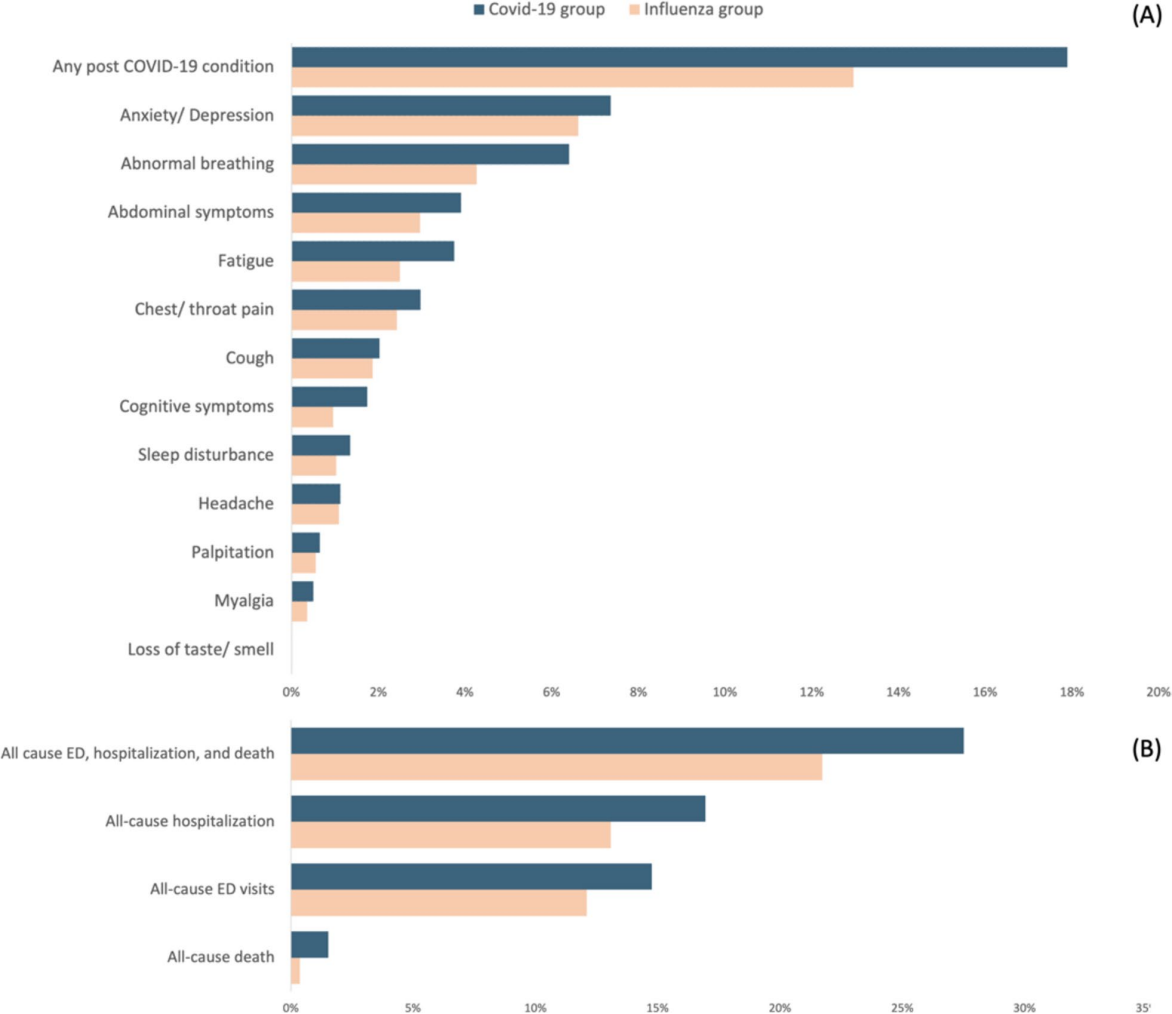
**A** Incidence of each post-COVID-19 condition within 90 to 180 days after COVID-19 diagnosis. **B** Incidence of all-cause emergency department (ED) visits, hospitalization and death within 90 to 180 days after COVID-19 diagnosis

Overall, the COVID-19 group had a higher incidence of any post-COVID-19 condition when compared with the influenza group (17.9% vs. 13.0%), with an HR of 1.398 (95% CI, 1.251–1.562) (Table S2). During the follow-up period, the study group had a higher risk of developing post-COVID conditions when compared with the control group (log-rank *p* < 0.05; Fig. 2). Specifically, anxiety/depression was the most common post-COVID-19 condition, followed by abnormal breathing, abdominal symptoms, fatigue, chest/throat pain, coughing, cognitive symptoms, sleep disturbance, headache, palpitations, myalgia, and loss of taste or smell (Fig. 3). Compared to the influenza group, the COVID-19 group had a significantly higher incidence of abnormal breathing (HR, 1.506; 95% CI, 1.246–1.822), abdominal symptoms (HR, 1.313; HR, 1.034–1.664), fatigue (HR, 1.486; 95% CI, 1.158–1.907), and cognitive symptoms (HR, 1.815; 95% CI, 1.235–2.668) (Table S4).

### Secondary outcomes

During the follow-up period, the COVID-19 group had a significantly higher risk of composite outcomes of all-cause ED visits, hospitalizations, and deaths when compared with the influenza group (27.5% vs. 21.7; HR, 1.303; 95% CI, 1.194–1.422) (Table S2). Specifically, the COVID-19 group had a higher risk of all-cause ED visits (14.8% vs. 12.1%; HR, 1.237; 95% CI, 1.098–1.393), hospitalizations (17.0% vs. 13.1%; HR, 1.302; 95% CI, 1.163–1.457), and mortality (1.5% vs. 0.4%; HR, 4.378; 95% CI, 2.573–7.449) (Table S2).

### Subgroup analysis

**Fig. 4:**
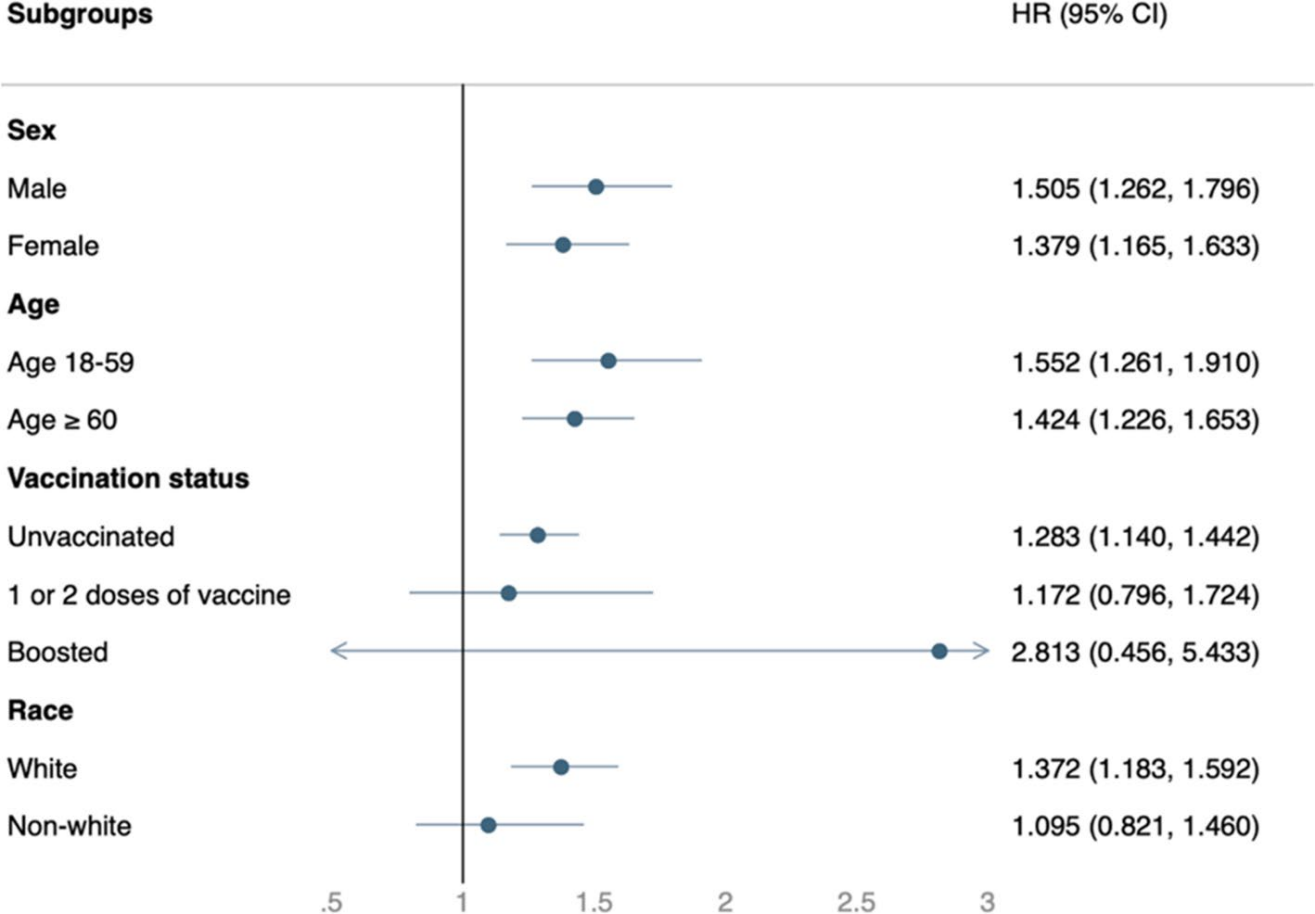
Subgroup analysis of the risk of post-COVID-19 condition between the COVID-19 group and Influenza group

**Fig. 5:**
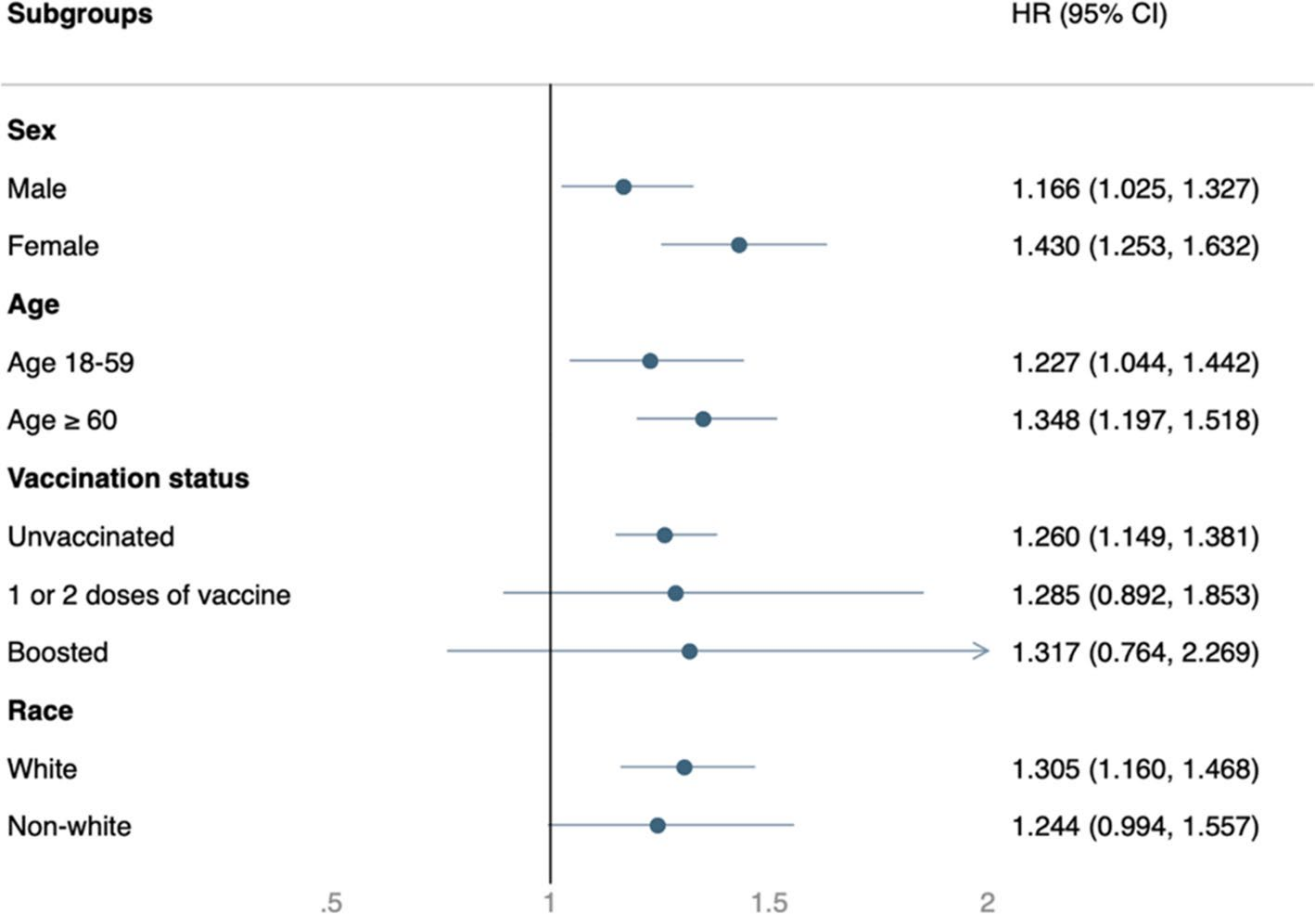
Subgroup analysis of the risk of all-cause emergency department (ED) visits, hospitalization and death between the COVID-19 group and Influenza group

In most subgroup analyses, the COVID-19 group was associated with a consistently and significantly higher risk of post-COVID conditions (Fig. 4) and composite clinical outcomes of all-cause ED visits, hospitalizations, and deaths (Fig. 5). Exceptions were those who had received the COVID-19 vaccination and non-Caucasian populations, in which only a non-significantly higher risk of post-acute sequelae in COVID-19 was found.

## Discussion

This retrospective study provides compelling evidence that patients who survive hospitalization for COVID-19 are at a higher risk of experiencing post-acute sequelae when compared to those with influenza. First, the COVID-19 group exhibited a higher risk of developing any post-COVID-19 condition when compared to the influenza group. Moreover, patients with COVID-19 had a heightened risk of experiencing specific symptoms such as abnormal breathing, abdominal symptoms, fatigue, and cognitive impairment. Second, beyond the post-COVID conditions, patients with COVID-19 also faced an elevated risk of all-cause ED visits, hospitalizations, and mortality following hospital discharge compared to those with influenza. Finally, these findings were consistent across various subgroups, reinforcing the notion that hospitalized patients with COVID-19 have increased susceptibility to post-acute sequelae. These results highlight the importance of monitoring and addressing the long-term consequences of SARS-CoV-2 infection, even after patients have survived hospitalization for acute COVID-19, emphasizing the need for ongoing vigilance from healthcare professionals.

Despite the downgrade of COVID-19 cases after May 2023, our findings suggest that it is crucial to continue monitoring the incidence of post-COVID conditions. Although significant progress has been made in controlling the spread of the virus and reducing the severity of acute infections, the potential long-term consequences of COVID-19 cannot be overlooked. The evolving understanding of the post-acute sequelae of SARS-CoV-2 infection and the persistence of symptoms among a subset of individuals necessitates continued monitoring to assess the true burden and impact of long COVID-19. By closely monitoring the incidence of post-COVID conditions, healthcare professionals, researchers, and policymakers can identify risk factors, develop appropriate interventions, and allocate resources to support individuals affected by this condition. Therefore, despite the decline in COVID-19 cases, vigilance and monitoring of the post-COVID-19 incidence remains imperative in addressing the long-term health effects of the pandemic.

The present findings contrast with prior research [19]. A population-based cohort study in Ontario, Canada showed that, except for a higher risk of venous thromboembolic disease compared with influenza (adjusted HR, 1.77; 95% CI, 1.36–2.31), hospitalization for COVID-19 was not associated with increased 1-year risks of developing ischemic and nonischemic cerebrovascular and cardiovascular disorders, neurological disorders, rheumatoid arthritis, or mental health conditions [19]. However, this study [19] focused on adult patients hospitalized for COVID-19 between April 1, 2020, and October 31, 2021; therefore, their findings may not be applicable to conditions after 2022, during which the Omicron variant was the predominant SARS-COV-2 and effective boosted vaccine and oral antiviral agents were implemented. This could explain the cause behind these conflicting findings. However, it's worth mentioning that while temporal and virological elements may help elucidate the differences in outcomes, other influential determinants should not be discounted. Factors such as population demographics, epidemiological conditions, and host genetic factors can exert substantial influences on the clinical presentations and consequences of COVID-19 [31, 32].

Here, a higher risk of post-acute sequelae following COVID-19 was consistently observed across most subgroup analyses, except for vaccinated patients. A potential explanation could be the protective effect of the COVID-19 vaccine against post-acute sequelae. A population-based analysis estimated that SARS-CoV-2 vaccine could decrease the prevalence of long COVID among U.S. adults by 20.9% (95% CI, -32.0%, -9.9%) and, from the analysis of 158 countries, by -15.7% (95% CI, -18.0%, -13.4%) among all who had COVID-19 [33]. Similarly, a meta-analysis reported that at least one vaccine dose was associated with a protective effect against long COVID (odds ratio, 0.539, 95% CI, 0.295–0.987) [34]. Moreover, vaccination remained effective against long COVD in patients either vaccinated before SARS-CoV-2 infection (risk ratio [RR], 0.82; 95% CI, 0.74–0.91) or vaccinated after SARS-CoV-2 infection (RR, 0.83; 95% CI, 0.74–0.92) [35]. Based on these findings, this study suggests that the COVID-19 vaccine can help reduce the risk of post-COVID conditions, since our findings that COVID-19 inpatients who received prior vaccination do not have an increased risk of post-acute sequela of SARS-CoV-2 infection.

### Strengths and limitations

This multinational and multicenter retrospective study included all adults hospitalized for COVID-19 or influenza and used a PSM approach to minimize confounding between the groups. The present findings were consistent across almost all subgroup analyses. In addition, this real-world study focused on the Omicron waves after 2022. Finally, it is difficult to determine whether the diagnosis of post-COVID conditions is related to preexisting underlying conditions or viral infections. To overcome this difficulty, we excluded patients with a history of post-COVID-19 symptoms prior to SARS-CoV-2 infection, which may have helped to make sure post-COVID conditions were newly developed following hospitalization. Therefore, this study provides updated information with robust evidence and high generalizability.

This study had several limitations. First, as an EHR-based study, we could not exclude residual errors, missing information, and inconsistencies. Incomplete documentation, coding variations, and data entry errors can introduce bias. EHR data may not represent the entire population as they primarily include individuals who seek medical care and have access to healthcare facilities. Additionally, it is important to understand that the absence of symptom reporting does not necessarily mean that symptoms have not occurred. However, this study focused on a specific group of patients who survived hospitalization. Thus, the associated selection bias was minimized. Second, although this study tried to adjust for most of the confounding variables between the study and control groups, residual factors that are not captured in the EHR, such as lifestyle or unmeasured comorbidities, may impact the outcomes of interest and potentially lead to biased results. Here, we did not assess the confounding effects of anti-COVID-19 treatments, such as anti-viral, systemic corticosteroid, or interleukin-6 blockade; however, these factors may affect the development of post-acute sequelae of SARS-CoV-2 infections [36–38]. Third, it is noteworthy that some post-COVID conditions may be mentioned more frequently in patients with COVID-19 compared to those with influenza, even if they are equally common, potentially due to heightened anticipation and awareness by both the patient and the healthcare provider. Forth, TrinetX is a database that encompasses a vast geographic span, including the Americas, Europe, the Middle East, Africa, and the Asia–Pacific regions, however, we cannot access to specific data on the contribution of individuals and HCOs from each country due to their privacy policy. We just knew that a significant portion of these collaborations is concentrated in the United States. Finally, this study focused on hospitalized patients with COVID-19 or influenza; however, most patients with COVID-19 or influenza did not require admission for treatment. Further studies are required to clarify these issues.

## Conclusions

This study highlights that hospitalized patients with COVID-19 may face a heightened risk of post-acute sequelae, including post-COVID conditions, all-cause ED visits, hospitalizations, and mortality, compared with influenza survivors within 90–180 days of SARS-CoV-2 infection. Despite the downgrading of the COVID-19 pandemic in many countries and treating it as influenza, this study serves as a reminder that SARS-CoV-2 infection may be associated with a greater likelihood of long-term complications. The potential impact of these sequelae on the health status and quality of life underscores the importance of clinicians maintaining vigilance regarding post-acute sequelae among patients who have survived hospitalization for COVID-19. By staying alert to these long-term risks and providing appropriate monitoring and care, healthcare professionals can better address the needs of patients in the aftermath of a SARS-CoV-2 infection.

### Supplementary Information

**Additional file 1: Table S1.** Query Criteria for Cohort (query name: covid_cnetwork). **Table S2.** Query Criteria for Cohort (query name:flu). **Table S3.** Outcome Definitions. **Table S4.** The hazard ratio and events number for comparing matched COVID-19 group and Influenza group for the primary composite outcome and its constituents.

## Data Availability

All data generated or analyzed during this study are included in this published article and will be available upon request to CCL.

## Abbreviations

COVID-19: Coronavirus disease 2019
CI: Confidence interval
ED: Emergency department
EHR: Electronic health record
HCO: Healthcare organization
HR: Hazard ratio
PSM: Propensity score-matching
SARS-CoV-2: Severe acute respiratory syndrome coronavirus 2
