# Supplementary material for: Comparison of post-acute sequelae following hospitalization for COVID-19 and influenza
Source: BMC Med. 2023 Dec 5;21:480. doi: 10.1186/s12916-023-03200-2 (PMC10696681; doi:10.1186/s12916-023-03200-2)
Supplement: Supplementary file 1 — Additional file 1: Table S1. Query Criteria for Cohort (query name: covid_cnetwork). Table S2. Query Criteria for Cohort (query name:flu). Table S3. Outcome Definitions. Table S4. The hazard ratio and events number for comparing matched COVID-19 group and Influenza group for the primary composite outcome and its constituents. [file 12916_2023_3200_MOESM1_ESM.docx]

**Table S1. Query Criteria for Cohort (query name: covid_cnetwork)**

|  | must have |  | demographics | Age | Age (at least 18 years) |
| --- | --- | --- | --- | --- | --- |
| Group 1 | | | | | |
|  | **visit** | | | | |
|  | must have |  | visit | TNX:Visit | Visit |
|  | number of occurrences | | Greater than or equal to 2 instances | | |
|  | date constraint | | The terms in this group occurred between Mar 1, 2020 and Jan 1, 2023 | | |
| Group 2 | | | | | |
|  | **Covid-19** | | | | |
|  | must have | any of | diagnosis | UMLS:ICD10CM:U07.1 | COVID-19 |
|  |  |  | diagnosis | UMLS:ICD10CM:J12.81 | Pneumonia due to SARS-associated coronavirus |
|  |  |  | diagnosis | UMLS:ICD10CM:J12.82 | Pneumonia due to coronavirus disease 2019 |
|  |  |  | laboratory | TNX:9088 | SARS coronavirus 2 and related RNA [Presence] |
|  |  |  | laboratory | UMLS:LNC:94309-2 | SARS-CoV-2 (COVID-19) RNA [Presence] in Specimen by NAA with probe detection |
|  |  |  | laboratory | UMLS:LNC:94500-6 | SARS-CoV-2 (COVID-19) RNA [Presence] in Respiratory specimen by NAA with probe detection |
|  |  |  | laboratory | UMLS:LNC:94502-2 | SARS-related coronavirus RNA [Presence] in Respiratory specimen by NAA with probe detection |
|  |  |  | laboratory | UMLS:LNC:95406-5 | SARS-CoV-2 (COVID-19) RNA [Presence] in Nose by NAA with probe detection |
|  |  |  | laboratory | UMLS:LNC:94565-9 | SARS-CoV-2 (COVID-19) RNA [Presence] in Nasopharynx by NAA with non-probe detection |
|  |  |  | laboratory | UMLS:LNC:95608-6 | SARS-CoV-2 (COVID-19) RNA [Presence] in Respiratory specimen by NAA with non-probe detection |
|  |  |  | laboratory | UMLS:LNC:94759-8 | SARS-CoV-2 (COVID-19) RNA [Presence] in Nasopharynx by NAA with probe detection |
|  |  |  | laboratory | UMLS:LNC:94845-5 | SARS-CoV-2 (COVID-19) RNA [Presence] in Saliva (oral fluid) by NAA with probe detection |
|  |  |  | laboratory | UMLS:LNC:96119-3 | SARS-CoV-2 (COVID-19) Ag [Presence] in Upper respiratory specimen by Immunoassay |
|  |  |  | laboratory | UMLS:LNC:94558-4 | SARS-CoV-2 (COVID-19) Ag [Presence] in Respiratory specimen by Rapid immunoassay |
|  | date constraint | | The terms in this group occurred between Jan 1, 2022 and Jan 1, 2023 | | |
| Group 3 | | | | | |
|  | **Group 3A Covid-19** | | | | |
|  | must have | any of | diagnosis | UMLS:ICD10CM:U07.1 | COVID-19 |
|  |  |  | diagnosis | UMLS:ICD10CM:J12.81 | Pneumonia due to SARS-associated coronavirus |
|  |  |  | diagnosis | UMLS:ICD10CM:J12.82 | Pneumonia due to coronavirus disease 2019 |
|  |  |  | laboratory | TNX:9088 | SARS coronavirus 2 and related RNA [Presence] |
|  |  |  | laboratory | UMLS:LNC:94309-2 | SARS-CoV-2 (COVID-19) RNA [Presence] in Specimen by NAA with probe detection |
|  |  |  | laboratory | UMLS:LNC:94500-6 | SARS-CoV-2 (COVID-19) RNA [Presence] in Respiratory specimen by NAA with probe detection |
|  |  |  | laboratory | UMLS:LNC:94502-2 | SARS-related coronavirus RNA [Presence] in Respiratory specimen by NAA with probe detection |
|  |  |  | laboratory | UMLS:LNC:95406-5 | SARS-CoV-2 (COVID-19) RNA [Presence] in Nose by NAA with probe detection |
|  |  |  | laboratory | UMLS:LNC:94565-9 | SARS-CoV-2 (COVID-19) RNA [Presence] in Nasopharynx by NAA with non-probe detection |
|  |  |  | laboratory | UMLS:LNC:95608-6 | SARS-CoV-2 (COVID-19) RNA [Presence] in Respiratory specimen by NAA with non-probe detection |
|  |  |  | laboratory | UMLS:LNC:94759-8 | SARS-CoV-2 (COVID-19) RNA [Presence] in Nasopharynx by NAA with probe detection |
|  |  |  | laboratory | UMLS:LNC:94845-5 | SARS-CoV-2 (COVID-19) RNA [Presence] in Saliva (oral fluid) by NAA with probe detection |
|  |  |  | laboratory | UMLS:LNC:96119-3 | SARS-CoV-2 (COVID-19) Ag [Presence] in Upper respiratory specimen by Immunoassay |
|  |  |  | laboratory | UMLS:LNC:94558-4 | SARS-CoV-2 (COVID-19) Ag [Presence] in Respiratory specimen by Rapid immunoassay |
|  | date constraint | | The terms in this group occurred at any time | | |
|  | event relationship | | Any instance of died occurred within 2 days before or up to 10 days after the first instance of Covid-19 | | |
|  | **Group 3B died** | | | | |
|  | must have | any of | visit | UMLS:HL7V3.0:VisitType:ACUTE | Visit: Inpatient Acute |
|  |  |  | visit | UMLS:HL7V3.0:VisitType:IMP | Visit: Inpatient Encounter |
|  |  |  | visit | UMLS:HL7V3.0:VisitType:NONAC | Visit: Inpatient Non-acute |
|  |  |  | visit | UMLS:HL7V3.0:VisitType:OBSENC | Visit: Observation Encounter |
|  |  |  | visit | UMLS:HL7V3.0:VisitType:SS | Visit: Short Stay |
|  |  |  | procedure | UMLS:CPT:1013659 | Hospital Inpatient Services |
|  |  |  | procedure | UMLS:CPT:1013729 | Critical Care Services |
|  |  |  | procedure | UMLS:CPT:1013699 | Inpatient Consultations |
|  |  |  | procedure | UMLS:CPT:1013660 | Initial Hospital Care |
| Group 4 | | | | | |
|  | **Group 4A Covid-19** | | | | |
|  | must have | any of | diagnosis | UMLS:ICD10CM:U07.1 | COVID-19 |
|  |  |  | diagnosis | UMLS:ICD10CM:J12.81 | Pneumonia due to SARS-associated coronavirus |
|  |  |  | diagnosis | UMLS:ICD10CM:J12.82 | Pneumonia due to coronavirus disease 2019 |
|  |  |  | laboratory | TNX:9088 | SARS coronavirus 2 and related RNA [Presence] |
|  |  |  | laboratory | UMLS:LNC:94309-2 | SARS-CoV-2 (COVID-19) RNA [Presence] in Specimen by NAA with probe detection |
|  |  |  | laboratory | UMLS:LNC:94500-6 | SARS-CoV-2 (COVID-19) RNA [Presence] in Respiratory specimen by NAA with probe detection |
|  |  |  | laboratory | UMLS:LNC:94502-2 | SARS-related coronavirus RNA [Presence] in Respiratory specimen by NAA with probe detection |
|  |  |  | laboratory | UMLS:LNC:95406-5 | SARS-CoV-2 (COVID-19) RNA [Presence] in Nose by NAA with probe detection |
|  |  |  | laboratory | UMLS:LNC:94565-9 | SARS-CoV-2 (COVID-19) RNA [Presence] in Nasopharynx by NAA with non-probe detection |
|  |  |  | laboratory | UMLS:LNC:95608-6 | SARS-CoV-2 (COVID-19) RNA [Presence] in Respiratory specimen by NAA with non-probe detection |
|  |  |  | laboratory | UMLS:LNC:94759-8 | SARS-CoV-2 (COVID-19) RNA [Presence] in Nasopharynx by NAA with probe detection |
|  |  |  | laboratory | UMLS:LNC:94845-5 | SARS-CoV-2 (COVID-19) RNA [Presence] in Saliva (oral fluid) by NAA with probe detection |
|  |  |  | laboratory | UMLS:LNC:96119-3 | SARS-CoV-2 (COVID-19) Ag [Presence] in Upper respiratory specimen by Immunoassay |
|  |  |  | laboratory | UMLS:LNC:94558-4 | SARS-CoV-2 (COVID-19) Ag [Presence] in Respiratory specimen by Rapid immunoassay |
|  | date constraint | | The terms in this group occurred at any time | | |
|  | event relationship | | Any instance of Group 4B occurred within 1 day before or up to 5 days after the first instance of Covid-19 | | |
|  | **Group 4B** | | | | |
|  | must have | any of | medication | NLM:RXNORM:2587901 | molnupiravir |
|  |  |  | medication | NLM:RXNORM:2587892 | nirmatrelvir |
|  |  |  | medication | NLM:RXNORM:2284718 | remdesivir |
|  |  |  | medication | NLM:RXNORM:85762 | ritonavir |
| Group 5 | | | | | |
|  | **Group 5A Covid-19** | | | | |
|  | must have | any of | diagnosis | UMLS:ICD10CM:U07.1 | COVID-19 |
|  |  |  | diagnosis | UMLS:ICD10CM:J12.81 | Pneumonia due to SARS-associated coronavirus |
|  |  |  | diagnosis | UMLS:ICD10CM:J12.82 | Pneumonia due to coronavirus disease 2019 |
|  |  |  | laboratory | TNX:9088 | SARS coronavirus 2 and related RNA [Presence] |
|  |  |  | laboratory | UMLS:LNC:94309-2 | SARS-CoV-2 (COVID-19) RNA [Presence] in Specimen by NAA with probe detection |
|  |  |  | laboratory | UMLS:LNC:94500-6 | SARS-CoV-2 (COVID-19) RNA [Presence] in Respiratory specimen by NAA with probe detection |
|  |  |  | laboratory | UMLS:LNC:94502-2 | SARS-related coronavirus RNA [Presence] in Respiratory specimen by NAA with probe detection |
|  |  |  | laboratory | UMLS:LNC:95406-5 | SARS-CoV-2 (COVID-19) RNA [Presence] in Nose by NAA with probe detection |
|  |  |  | laboratory | UMLS:LNC:94565-9 | SARS-CoV-2 (COVID-19) RNA [Presence] in Nasopharynx by NAA with non-probe detection |
|  |  |  | laboratory | UMLS:LNC:95608-6 | SARS-CoV-2 (COVID-19) RNA [Presence] in Respiratory specimen by NAA with non-probe detection |
|  |  |  | laboratory | UMLS:LNC:94759-8 | SARS-CoV-2 (COVID-19) RNA [Presence] in Nasopharynx by NAA with probe detection |
|  |  |  | laboratory | UMLS:LNC:94845-5 | SARS-CoV-2 (COVID-19) RNA [Presence] in Saliva (oral fluid) by NAA with probe detection |
|  |  |  | laboratory | UMLS:LNC:96119-3 | SARS-CoV-2 (COVID-19) Ag [Presence] in Upper respiratory specimen by Immunoassay |
|  |  |  | laboratory | UMLS:LNC:94558-4 | SARS-CoV-2 (COVID-19) Ag [Presence] in Respiratory specimen by Rapid immunoassay |
|  | date constraint | | The terms in this group occurred at any time | | |
|  | event relationship | | Any instance of long covid occurred within 1 year on or before the first instance of Covid-19 | | |
|  | **Group 5B long covid** | | | | |
|  | cannot have |  | diagnosis | UMLS:ICD10CM:R07 | Pain in throat and chest |
|  |  | or | diagnosis | UMLS:ICD10CM:R06 | Abnormalities of breathing |
|  |  | or | diagnosis | UMLS:ICD10CM:R10 | Abdominal and pelvic pain |
|  |  | or | diagnosis | UMLS:ICD10CM:R19.4 | Change in bowel habit |
|  |  | or | diagnosis | UMLS:ICD10CM:R19.7 | Diarrhea, unspecified |
|  |  | or | diagnosis | UMLS:ICD10CM:G93.3 | Postviral and related fatigue syndromes |
|  |  | or | diagnosis | UMLS:ICD10CM:R53 | Malaise and fatigue |
|  |  | or | diagnosis | UMLS:ICD10CM:R51 | Headache |
|  |  | or | diagnosis | UMLS:ICD10CM:G43 | Migraine |
|  |  | or | diagnosis | UMLS:ICD10CM:G44 | Other headache syndromes |
|  |  | or | diagnosis | UMLS:ICD10CM:R40 | Somnolence, stupor and coma |
|  |  | or | diagnosis | UMLS:ICD10CM:R41 | Other symptoms and signs involving cognitive functions and awareness |
|  |  | or | diagnosis | UMLS:ICD10CM:R48 | Dyslexia and other symbolic dysfunctions, not elsewhere classified |
|  |  | or | diagnosis | UMLS:ICD10CM:G93.40 | Encephalopathy, unspecified |
|  |  | or | diagnosis | UMLS:ICD10CM:G31.84 | Mild cognitive impairment of uncertain or unknown etiology |
|  |  | or | diagnosis | UMLS:ICD10CM:G30 | Alzheimer's disease |
|  |  | or | diagnosis | UMLS:ICD10CM:G31.0 | Frontotemporal dementia |
|  |  | or | diagnosis | UMLS:ICD10CM:G31.83 | Neurocognitive disorder with Lewy bodies |
|  |  | or | diagnosis | UMLS:ICD10CM:F01 | Vascular dementia |
|  |  | or | diagnosis | UMLS:ICD10CM:F02 | Dementia in other diseases classified elsewhere |
|  |  | or | diagnosis | UMLS:ICD10CM:F03 | Unspecified dementia |
|  |  | or | diagnosis | UMLS:ICD10CM:F05 | Delirium due to known physiological condition |
|  |  | or | diagnosis | UMLS:ICD10CM:F06.8 | Other specified mental disorders due to known physiological condition |
|  |  | or | diagnosis | UMLS:ICD10CM:M79.1 | Myalgia |
|  |  | or | diagnosis | UMLS:ICD10CM:M60 | Myositis |
|  |  | or | diagnosis | UMLS:ICD10CM:R43.8 | Other disturbances of smell and taste |
|  |  | or | diagnosis | UMLS:ICD10CM:G47.9 | Sleep disorder, unspecified |
|  |  | or | diagnosis | UMLS:ICD10CM:R05 | Cough |
|  |  | or | diagnosis | UMLS:ICD10CM:R00.2 | Palpitations |

### **Table S2. Query Criteria for Cohort (query name:flu)**

| \| Ungrouped terms \| \| \| \| \| \| \| --- \| --- \| --- \| --- \| --- \| --- \| \|  \| must have \|  \| demographics \| Age \| Age (at least 18 years) \| \| Group 1 \| \| \| \| \| \| \|  \| **visit** \| \| \| \| \| \|  \| must have \|  \| visit \| TNX:Visit \| Visit \| \|  \| number of occurrences \| \| Greater than or equal to 2 instances \| \| \| \|  \| date constraint \| \| The terms in this group occurred between Mar 1, 2020 and Jan 1, 2023 \| \| \| \| Group 2 \| \| \| \| \| \| \|  \| **Covid-19** \| \| \| \| \| \|  \| must have \| any of \| diagnosis \| UMLS:ICD10CM:J09 \| Influenza due to certain identified influenza viruses \| \|  \|  \|  \| diagnosis \| UMLS:ICD10CM:J10 \| Influenza due to other identified influenza virus \| \|  \|  \|  \| diagnosis \| UMLS:ICD10CM:J11 \| Influenza due to unidentified influenza virus \| \|  \| date constraint \| \| The terms in this group occurred at any time \| \| \| \| Group 3 \| \| \| \| \| \| \|  \| **Group 3A Covid-19** \| \| \| \| \| \|  \| must have \| any of \| diagnosis \| UMLS:ICD10CM:J09 \| Influenza due to certain identified influenza viruses \| \|  \|  \|  \| diagnosis \| UMLS:ICD10CM:J10 \| Influenza due to other identified influenza virus \| \|  \|  \|  \| diagnosis \| UMLS:ICD10CM:J11 \| Influenza due to unidentified influenza virus \| \|  \| date constraint \| \| The terms in this group occurred at any time \| \| \| \|  \| event relationship \| \| Any instance of hos occurred within 2 days before or up to 1 month after the first instance of Covid-19 \| \| \| \|  \| **Group 3B hos** \| \| \| \| \| \|  \| must have \| any of \| visit \| UMLS:HL7V3.0:VisitType:ACUTE \| Visit: Inpatient Acute \| \|  \|  \|  \| visit \| UMLS:HL7V3.0:VisitType:IMP \| Visit: Inpatient Encounter \| \|  \|  \|  \| visit \| UMLS:HL7V3.0:VisitType:NONAC \| Visit: Inpatient Non-acute \| \|  \|  \|  \| visit \| UMLS:HL7V3.0:VisitType:OBSENC \| Visit: Observation Encounter \| \|  \|  \|  \| visit \| UMLS:HL7V3.0:VisitType:SS \| Visit: Short Stay \| \|  \|  \|  \| procedure \| UMLS:CPT:1013659 \| Hospital Inpatient Services \| \|  \|  \|  \| procedure \| UMLS:CPT:1013729 \| Critical Care Services \| \|  \|  \|  \| procedure \| UMLS:CPT:1013699 \| Inpatient Consultations \| \|  \|  \|  \| procedure \| UMLS:CPT:1013660 \| Initial Hospital Care \| \| Group 4 \| \| \| \| \| \| \|  \| **Group 4A Covid-19** \| \| \| \| \| \|  \| must have \| any of \| diagnosis \| UMLS:ICD10CM:J09 \| Influenza due to certain identified influenza viruses \| \|  \|  \|  \| diagnosis \| UMLS:ICD10CM:J10 \| Influenza due to other identified influenza virus \| \|  \|  \|  \| diagnosis \| UMLS:ICD10CM:J11 \| Influenza due to unidentified influenza virus \| \|  \| date constraint \| \| The terms in this group occurred at any time \| \| \| \|  \| event relationship \| \| Any instance of Group 4B occurred within 1 day before or up to 5 days after the first instance of Covid-19 \| \| \| \|  \| **Group 4B** \| \| \| \| \| \|  \| must have \|  \| medication \| NLM:RXNORM:260101 \| oseltamivir \| \| Group 5 \| \| \| \| \| \| \|  \| **Group 5A Covid-19** \| \| \| \| \| \|  \| must have \| any of \| diagnosis \| UMLS:ICD10CM:J09 \| Influenza due to certain identified influenza viruses \| \|  \|  \|  \| diagnosis \| UMLS:ICD10CM:J10 \| Influenza due to other identified influenza virus \| \|  \|  \|  \| diagnosis \| UMLS:ICD10CM:J11 \| Influenza due to unidentified influenza virus \| \|  \| date constraint \| \| The terms in this group occurred at any time \| \| \| \|  \| event relationship \| \| Any instance of long covid occurred within 1 year on or before the first instance of Covid-19 \| \| \| \|  \| **Group 5B long covid** \| \| \| \| \| \|  \| cannot have \|  \| diagnosis \| UMLS:ICD10CM:R07 \| Pain in throat and chest \| \|  \|  \| or \| diagnosis \| UMLS:ICD10CM:R06 \| Abnormalities of breathing \| \|  \|  \| or \| diagnosis \| UMLS:ICD10CM:R10 \| Abdominal and pelvic pain \| \|  \|  \| or \| diagnosis \| UMLS:ICD10CM:R19.4 \| Change in bowel habit \| \|  \|  \| or \| diagnosis \| UMLS:ICD10CM:R19.7 \| Diarrhea, unspecified \| \|  \|  \| or \| diagnosis \| UMLS:ICD10CM:G93.3 \| Postviral and related fatigue syndromes \| \|  \|  \| or \| diagnosis \| UMLS:ICD10CM:R53 \| Malaise and fatigue \| \|  \|  \| or \| diagnosis \| UMLS:ICD10CM:R51 \| Headache \| \|  \|  \| or \| diagnosis \| UMLS:ICD10CM:G43 \| Migraine \| \|  \|  \| or \| diagnosis \| UMLS:ICD10CM:G44 \| Other headache syndromes \| \|  \|  \| or \| diagnosis \| UMLS:ICD10CM:R40 \| Somnolence, stupor and coma \| \|  \|  \| or \| diagnosis \| UMLS:ICD10CM:R41 \| Other symptoms and signs involving cognitive functions and awareness \| \|  \|  \| or \| diagnosis \| UMLS:ICD10CM:R48 \| Dyslexia and other symbolic dysfunctions, not elsewhere classified \| \|  \|  \| or \| diagnosis \| UMLS:ICD10CM:G93.40 \| Encephalopathy, unspecified \| \|  \|  \| or \| diagnosis \| UMLS:ICD10CM:G31.84 \| Mild cognitive impairment of uncertain or unknown etiology \| \|  \|  \| or \| diagnosis \| UMLS:ICD10CM:G30 \| Alzheimer's disease \| \|  \|  \| or \| diagnosis \| UMLS:ICD10CM:G31.0 \| Frontotemporal dementia \| \|  \|  \| or \| diagnosis \| UMLS:ICD10CM:G31.83 \| Neurocognitive disorder with Lewy bodies \| \|  \|  \| or \| diagnosis \| UMLS:ICD10CM:F01 \| Vascular dementia \| \|  \|  \| or \| diagnosis \| UMLS:ICD10CM:F02 \| Dementia in other diseases classified elsewhere \| \|  \|  \| or \| diagnosis \| UMLS:ICD10CM:F03 \| Unspecified dementia \| \|  \|  \| or \| diagnosis \| UMLS:ICD10CM:F05 \| Delirium due to known physiological condition \| \|  \|  \| or \| diagnosis \| UMLS:ICD10CM:F06.8 \| Other specified mental disorders due to known physiological condition \| \|  \|  \| or \| diagnosis \| UMLS:ICD10CM:M79.1 \| Myalgia \| \|  \|  \| or \| diagnosis \| UMLS:ICD10CM:M60 \| Myositis \| \|  \|  \| or \| diagnosis \| UMLS:ICD10CM:R43.8 \| Other disturbances of smell and taste \| \|  \|  \| or \| diagnosis \| UMLS:ICD10CM:G47.9 \| Sleep disorder, unspecified \| \|  \|  \| or \| diagnosis \| UMLS:ICD10CM:R05 \| Cough \| \|  \|  \| or \| diagnosis \| UMLS:ICD10CM:R00.2 \| Palpitations \| |
| --- | --- | --- | --- | --- | --- | --- | --- | --- | --- | --- | --- | --- | --- | --- | --- | --- | --- | --- | --- | --- | --- | --- | --- | --- | --- | --- | --- | --- | --- | --- | --- | --- | --- | --- | --- | --- | --- | --- | --- | --- | --- | --- | --- | --- | --- | --- | --- | --- | --- | --- | --- | --- | --- | --- | --- | --- | --- | --- | --- | --- | --- | --- | --- | --- | --- | --- | --- | --- | --- | --- | --- | --- | --- | --- | --- | --- | --- | --- | --- | --- | --- | --- | --- | --- | --- | --- | --- | --- | --- | --- | --- | --- | --- | --- | --- | --- | --- | --- | --- | --- | --- | --- | --- | --- | --- | --- | --- | --- | --- | --- | --- | --- | --- | --- | --- | --- | --- | --- | --- | --- | --- | --- | --- | --- | --- | --- | --- | --- | --- | --- | --- | --- | --- | --- | --- | --- | --- | --- | --- | --- | --- | --- | --- | --- | --- | --- | --- | --- | --- | --- | --- | --- | --- | --- | --- | --- | --- | --- | --- | --- | --- | --- | --- | --- | --- | --- | --- | --- | --- | --- | --- | --- | --- | --- | --- | --- | --- | --- | --- | --- | --- | --- | --- | --- | --- | --- | --- | --- | --- | --- | --- | --- | --- | --- | --- | --- | --- | --- | --- | --- | --- | --- | --- | --- | --- | --- | --- | --- | --- | --- | --- | --- | --- | --- | --- | --- | --- | --- | --- | --- | --- | --- | --- | --- | --- | --- | --- | --- | --- | --- | --- | --- | --- | --- | --- | --- | --- | --- | --- | --- | --- | --- | --- | --- | --- | --- | --- | --- | --- | --- | --- | --- | --- | --- | --- | --- | --- | --- | --- | --- | --- | --- | --- | --- | --- | --- | --- | --- | --- | --- | --- | --- | --- | --- | --- | --- | --- | --- | --- | --- | --- | --- | --- | --- | --- | --- | --- | --- | --- | --- | --- | --- | --- | --- | --- | --- | --- | --- | --- | --- | --- | --- | --- | --- | --- | --- | --- | --- | --- | --- | --- | --- | --- | --- | --- | --- | --- | --- | --- | --- | --- | --- | --- | --- | --- | --- | --- | --- | --- | --- | --- | --- | --- | --- | --- | --- | --- | --- | --- | --- | --- | --- | --- | --- | --- | --- | --- | --- | --- | --- | --- | --- | --- | --- | --- | --- | --- | --- | --- | --- | --- | --- | --- | --- | --- | --- | --- | --- | --- | --- | --- | --- | --- | --- | --- | --- | --- | --- | --- | --- | --- | --- | --- | --- | --- | --- | --- | --- | --- | --- | --- | --- | --- | --- | --- | --- | --- | --- | --- | --- | --- | --- | --- | --- | --- | --- | --- | --- | --- | --- | --- | --- | --- | --- | --- | --- | --- | --- | --- | --- | --- | --- | --- | --- | --- | --- | --- | --- | --- | --- | --- | --- | --- | --- | --- | --- | --- | --- | --- | --- | --- | --- | --- | --- | --- | --- | --- | --- | --- | --- | --- | --- | --- | --- | --- | --- |

### **Table S3.** Outcome Definitions

| main | | | | |
| --- | --- | --- | --- | --- |
|  | **Outcome definition** | | | |
|  | | Diagnosis | UMLS:ICD10CM:R07 | Pain in throat and chest |
|  | | Diagnosis | UMLS:ICD10CM:R06 | Abnormalities of breathing |
|  | | Diagnosis | UMLS:ICD10CM:R10 | Abdominal and pelvic pain |
|  | | Diagnosis | UMLS:ICD10CM:R19.4 | Change in bowel habit |
|  | | Diagnosis | UMLS:ICD10CM:R19.7 | Diarrhea, unspecified |
|  | | Diagnosis | UMLS:ICD10CM:G93.3 | Postviral and related fatigue syndromes |
|  | | Diagnosis | UMLS:ICD10CM:R53 | Malaise and fatigue |
|  | | Diagnosis | UMLS:ICD10CM:R51 | Headache |
|  | | Diagnosis | UMLS:ICD10CM:G43 | Migraine |
|  | | Diagnosis | UMLS:ICD10CM:G44 | Other headache syndromes |
|  | | Diagnosis | UMLS:ICD10CM:R40 | Somnolence, stupor and coma |
|  | | Diagnosis | UMLS:ICD10CM:R48 | Dyslexia and other symbolic dysfunctions, not elsewhere classified |
|  | | Diagnosis | UMLS:ICD10CM:F01 | Vascular dementia |
|  | | Diagnosis | UMLS:ICD10CM:F02 | Dementia in other diseases classified elsewhere |
|  | | Diagnosis | UMLS:ICD10CM:F03 | Unspecified dementia |
|  | | Diagnosis | UMLS:ICD10CM:G31.83 | Neurocognitive disorder with Lewy bodies |
|  | | Diagnosis | UMLS:ICD10CM:G31.84 | Mild cognitive impairment of uncertain or unknown etiology |
|  | | Diagnosis | UMLS:ICD10CM:G93.40 | Encephalopathy, unspecified |
|  | | Diagnosis | UMLS:ICD10CM:G30 | Alzheimer's disease |
|  | | Diagnosis | UMLS:ICD10CM:G31.0 | Frontotemporal dementia |
|  | | Diagnosis | UMLS:ICD10CM:F05 | Delirium due to known physiological condition |
|  | | Diagnosis | UMLS:ICD10CM:F06.8 | Other specified mental disorders due to known physiological condition |
|  | | Diagnosis | UMLS:ICD10CM:M79.1 | Myalgia |
|  | | Diagnosis | UMLS:ICD10CM:M60 | Myositis |
|  | | Diagnosis | UMLS:ICD10CM:R43.8 | Other disturbances of smell and taste |
|  | | Diagnosis | UMLS:ICD10CM:G47.0 | Insomnia |
|  | | Diagnosis | UMLS:ICD10CM:G47.9 | Sleep disorder, unspecified |
|  | | Diagnosis | UMLS:ICD10CM:R05 | Cough |
|  | | Diagnosis | UMLS:ICD10CM:R00.2 | Palpitations |
|  | **Settings for the performed analyses** | | | |
|  | | Kaplan - Meier survival analysis | | including patients with outcome prior to the time window |
| 1) Chest/Throat pain: | | | | |
|  | **Outcome definition** | | | |
|  | | Diagnosis | UMLS:ICD10CM:R07 | Pain in throat and chest |
|  | **Settings for the performed analyses** | | | |
|  | | Kaplan - Meier survival analysis | | including patients with outcome prior to the time window |
| Abnormal breathing | | | | |
|  | **Outcome definition** | | | |
|  | | Diagnosis | UMLS:ICD10CM:R06 | Abnormalities of breathing |
|  | **Settings for the performed analyses** | | | |
|  | | Kaplan - Meier survival analysis | | including patients with outcome prior to the time window |
| Abdominal symptoms | | | | |
|  | **Outcome definition** | | | |
|  | | Diagnosis | UMLS:ICD10CM:R10 | Abdominal and pelvic pain |
|  | | Diagnosis | UMLS:ICD10CM:R19.4 | Change in bowel habit |
|  | | Diagnosis | UMLS:ICD10CM:R19.7 | Diarrhea, unspecified |
|  | **Settings for the performed analyses** | | | |
|  | | Kaplan - Meier survival analysis | | including patients with outcome prior to the time window |
| Fatigue: | | | | |
|  | **Outcome definition** | | | |
|  | | Diagnosis | UMLS:ICD10CM:G93.3 | Postviral and related fatigue syndromes |
|  | | Diagnosis | UMLS:ICD10CM:R53 | Malaise and fatigue |
|  | **Settings for the performed analyses** | | | |
|  | | Kaplan - Meier survival analysis | | including patients with outcome prior to the time window |
| Anxiety/Depression | | | | |
|  | **Outcome definition** | | | |
|  | | Diagnosis | UMLS:ICD10CM:F30-F39 | Mood [affective] disorders |
|  | | Diagnosis | UMLS:ICD10CM:F40-F48 | Anxiety, dissociative, stress-related, somatoform and other nonpsychotic mental disorders |
|  | **Settings for the performed analyses** | | | |
|  | | Kaplan - Meier survival analysis | | including patients with outcome prior to the time window |
| headache | | | | |
|  | **Outcome definition** | | | |
|  | | Diagnosis | UMLS:ICD10CM:R51 | Headache |
|  | | Diagnosis | UMLS:ICD10CM:G43 | Migraine |
|  | | Diagnosis | UMLS:ICD10CM:G44 | Other headache syndromes |
|  | **Settings for the performed analyses** | | | |
|  | | Kaplan - Meier survival analysis | | including patients with outcome prior to the time window |
| Cognitive symptoms | | | | |
|  | **Outcome definition** | | | |
|  | | Diagnosis | UMLS:ICD10CM:R40 | Somnolence, stupor and coma |
|  | | Diagnosis | UMLS:ICD10CM:R48 | Dyslexia and other symbolic dysfunctions, not elsewhere classified |
|  | | Diagnosis | UMLS:ICD10CM:G93.40 | Encephalopathy, unspecified |
|  | | Diagnosis | UMLS:ICD10CM:G31.84 | Mild cognitive impairment of uncertain or unknown etiology |
|  | | Diagnosis | UMLS:ICD10CM:G30 | Alzheimer's disease |
|  | | Diagnosis | UMLS:ICD10CM:G31.0 | Frontotemporal dementia |
|  | | Diagnosis | UMLS:ICD10CM:G31.83 | Neurocognitive disorder with Lewy bodies |
|  | | Diagnosis | UMLS:ICD10CM:F01 | Vascular dementia |
|  | | Diagnosis | UMLS:ICD10CM:F02 | Dementia in other diseases classified elsewhere |
|  | | Diagnosis | UMLS:ICD10CM:F03 | Unspecified dementia |
|  | | Diagnosis | UMLS:ICD10CM:F05 | Delirium due to known physiological condition |
|  | | Diagnosis | UMLS:ICD10CM:F06.8 | Other specified mental disorders due to known physiological condition |
|  | **Settings for the performed analyses** | | | |
|  | | Kaplan - Meier survival analysis | | including patients with outcome prior to the time window |
| 9) Myalgia | | | | |
|  | **Outcome definition** | | | |
|  | | Diagnosis | UMLS:ICD10CM:M79.1 | Myalgia |
|  | | Diagnosis | UMLS:ICD10CM:M60 | Myositis |
|  | **Settings for the performed analyses** | | | |
|  | | Kaplan - Meier survival analysis | | including patients with outcome prior to the time window |
| Loss of taste/smell: | | | | |
|  | **Outcome definition** | | | |
|  | | Diagnosis | UMLS:ICD10CM:R43.8 | Other disturbances of smell and taste |
|  | **Settings for the performed analyses** | | | |
|  | | Kaplan - Meier survival analysis | | including patients with outcome prior to the time window |
| Sleep disturbance: | | | | |
|  | **Outcome definition** | | | |
|  | | Diagnosis | UMLS:ICD10CM:G47.0 | Insomnia |
|  | | Diagnosis | UMLS:ICD10CM:G47.9 | Sleep disorder, unspecified |
|  | **Settings for the performed analyses** | | | |
|  | | Kaplan - Meier survival analysis | | including patients with outcome prior to the time window |
| Cough: | | | | |
|  | **Outcome definition** | | | |
|  | | Diagnosis | UMLS:ICD10CM:R05 | Cough |
|  | **Settings for the performed analyses** | | | |
|  | | Kaplan - Meier survival analysis | | including patients with outcome prior to the time window |
| Palpitation | | | | |
|  | **Outcome definition** | | | |
|  | | Diagnosis | UMLS:ICD10CM:R00.2 | Palpitations |
|  | **Settings for the performed analyses** | | | |
|  | | Kaplan - Meier survival analysis | | including patients with outcome prior to the time window |
| 15) U09.9 Post COVID-19 condition | | | | |
|  | **Outcome definition** | | | |
|  | | Diagnosis | UMLS:ICD10CM:U09.9 | Post COVID-19 condition, unspecified |
|  | **Settings for the performed analyses** | | | |
|  | | Kaplan - Meier survival analysis | | including patients with outcome prior to the time window |
| all cause ER, hospitalization and death | | | | |
|  | **Outcome definition** | | | |
|  | | Visit | UMLS:HL7V3.0:VisitType:EMER | Visit: Emergency |
|  | | Visit | UMLS:HL7V3.0:VisitType:IMP | Visit: Inpatient Encounter |
|  | | Visit | UMLS:HL7V3.0:VisitType:NONAC | Visit: Inpatient Non-acute |
|  | | Visit | UMLS:HL7V3.0:VisitType:OBSENC | Visit: Observation Encounter |
|  | | Visit | UMLS:HL7V3.0:VisitType:SS | Visit: Short Stay |
|  | | Demographics | Deceased | Deceased |
|  | **Settings for the performed analyses** | | | |
|  | | Kaplan - Meier survival analysis | | including patients with outcome prior to the time window |
| ER | | | | |
|  | **Outcome definition** | | | |
|  | | Visit | UMLS:HL7V3.0:VisitType:EMER | Visit: Emergency |
|  | **Settings for the performed analyses** | | | |
|  | | Kaplan - Meier survival analysis | | including patients with outcome prior to the time window |
| Hospitalization | | | | |
|  | **Outcome definition** | | | |
|  | | Visit | UMLS:HL7V3.0:VisitType:IMP | Visit: Inpatient Encounter |
|  | | Visit | UMLS:HL7V3.0:VisitType:NONAC | Visit: Inpatient Non-acute |
|  | | Visit | UMLS:HL7V3.0:VisitType:OBSENC | Visit: Observation Encounter |
|  | | Visit | UMLS:HL7V3.0:VisitType:SS | Visit: Short Stay |
|  | **Settings for the performed analyses** | | | |
|  | | Kaplan - Meier survival analysis | | including patients with outcome prior to the time window |
| death | | | | |
|  | **Outcome definition** | | | |
|  | | Demographics | Deceased | Deceased |
|  | **Settings for the performed analyses** | | | |
|  | | Kaplan - Meier survival analysis | | including patients with outcome prior to the time window |

**Table S4. The hazard ratio and events number for comparing matched COVID-19 group and Influenza group for the primary composite outcome and its constituents.**

| Outcomes | Incidence of post-COVID-19 condition (%) | | Hazard ratio | (95%CI) | P value |
| --- | --- | --- | --- | --- | --- |
|  | COVID-19 group | Influenza group |  |  |  |
| **Any post COVID-19 condition** | 17.89 | 12.96 | 1.398 | (1.251, 1.562) | **<.001** |
| Chest/throat pain | 2.97 | 2.43 | 1.226 | (0.938, 1.601) | 0.135 |
| Abnormal breathing | 6.40 | 4.27 | 1.506 | (1.246, 1.822) | **<.001** |
| Abdominal symptoms | 3.91 | 2.96 | 1.313 | (1.037, 1.664) | **0.024** |
| Fatigue | 3.75 | 2.50 | 1.486 | (1.158, 1.907) | **0.002** |
| Anxiety/ Depression | 7.36 | 6.61 | 1.117 | (0.947, 1.319) | 0.189 |
| Headache | 1.12 | 1.09 | 1.002 | (0.659, 1.524) | 0.992 |
| Cognitive symptoms | 1.74 | 0.96 | 1.815 | (1.235, 2.668) | **0.002** |
| Myalgia | 0.50 | 0.36 | 1.397 | (0.710, 2.751) | 0.331 |
| Loss of taste/smell | 0 | 0 | - | - | - |
| Sleep disturbance | 1.35 | 1.03 | 1.292 | (0.862, 1.936) | 0.214 |
| Cough | 2.03 | 1.87 | 1.076 | (0.784, 1.477) | 0.651 |
| Palpitation | 0.65 | 0.56 | 1.144 | (0.649, 2.017) | 0.640 |
| **All cause ED visits, hospitalization, and death** | 27.50 | 21.72 | 1.303 | (1.194, 1.422) | **<.001** |
| All-cause ED visits | 14.75 | 12.10 | 1.237 | (1.098, 1.393) | **<.001** |
| All-cause hospitalization | 16.95 | 13.08 | 1.302 | (1.163, 1.457) | **<.001** |
| All-cause death | 1.53 | 0.36 | 4.378 | (2.573, 7.449) | **<.001** |
